# Isoprenylcysteine carboxylmethyltransferase is required for the impact of mutant KRAS on TAZ protein level and cancer cell self-renewal

**DOI:** 10.1038/s41388-020-1364-7

**Published:** 2020-06-19

**Authors:** Tin Fan Chai, Kanjoormana Aryan Manu, Patrick J. Casey, Mei Wang

**Affiliations:** 1 Program in Cancer and Stem Cell Biology, Duke-NUS Medical School, Singapore, 169857 Singapore; 2 Department of Biochemistry, National University of Singapore, Singapore, 117596 Singapore; 3 Department of Pharmacology and Cancer Biology, Duke University Medical Center, Durham, NC 27710 USA

**Keywords:** Cancer stem cells, Oncogenes, Growth factor signalling

## Abstract

Cancer stem cells possess the capacity for self-renewal and resistance to chemotherapy. It is therefore crucial to understand the molecular regulators of stemness in the quest to develop effective cancer therapies. TAZ is a transcription activator that promotes stem cell functions in post-development mammalian cells; suppression of TAZ activity reduces or eliminates cancer stemness in select cancers. Isoprenylcysteine carboxylmethyltransferase (ICMT) is the unique enzyme of the last step of posttranslational prenylation processing pathway that modifies several oncogenic proteins, including RAS. We found that suppression of ICMT results in reduced self-renewal/stemness in KRAS-driven pancreatic and breast cancer cells. Silencing of ICMT led to significant reduction of TAZ protein levels and loss of self-renewal ability, which could be reversed by overexpressing mutant KRAS, demonstrating the functional impact of ICMT modification on the ability of KRAS to control TAZ stability and function. Contrary to expectation, YAP protein levels appear to be much less susceptible than TAZ to the regulation by ICMT and KRAS, and YAP is less consequential in regulating stemness characteristics in these cells. Further, we found that the ICMT-dependent KRAS regulation of TAZ was mediated through RAF, but not PI3K, signaling. Functionally, we demonstrate that a signaling cascade from ICMT modification of KRAS to TAZ protein stability supports cancer cell self-renewal abilities in both in vitro and in vivo settings. In addition, studies using the proof-of-concept small molecule inhibitors of ICMT confirmed its role in regulating TAZ and self-renewal, demonstrating the potential utility of targeting ICMT to control aggressive KRAS-driven cancers.

## Introduction

Cancer stem cells (CSC) or tumor-initiating cells are thought to exist in all cancers, particularly the aggressive solid tumors such as pancreatic and metastatic breast cancers [1–3]. CSCs possess key features that include the ability of self-renewal, metastasis, and resistance to chemotherapies [1, 4–7], which are the main causes for mortality. In pancreatic cancers, for example, it has been reported that treatment with the frontline chemotherapy agent gemcitabine enriches the stem cells in the surviving population of tumor cells [8]. Similar enrichment of stem-like populations in breast cancers has been observed after doxorubicin treatment [9]. It remains a major challenge to develop effective targeted therapies that can eradicate CSCs.

The Hippo-YAP/TAZ signaling pathway is a complex network that includes kinases, transcription factors, and regulators that are essential in embryonic development [10–12]. The upstream components of this pathway consist primarily of an intracellular kinase cascade that suppresses the YAP/TAZ transcriptional activator functions involved in cell proliferation, survival, and migration [4, 10, 13–16]. In post-development organisms, activation of YAP/TAZ function confers cells with stem-like characteristics that leads to uncontrolled cell proliferation and tumorigenesis [17–19]. Over-activation of YAP/TAZ also contributes to the treatment failure of various solid tumors [20–25]. Hence, inhibiting YAP/TAZ function could potentially improve outcomes in relevant cancers. While genetic analyses suggest that direct mutations of the Hippo-YAP/TAZ pathway proteins are rare in human cancers, upregulation of YAP/TAZ activities, mostly the result of cross-talk from other major signaling pathways, are frequently observed [4, 12, 19, 26–28]. Identification of novel regulators, and elucidation of the mechanism of action of known regulators, of this pathway will not only add new dimensions of understanding but also offer new opportunities for therapeutic development.

KRAS is one of the most mutated oncogenes in human cancers [29–33]. As major regulators of cancer stemness, positive interactions between KRAS and YAP/TAZ have been observed in multiple cancers [34–37]. An interesting observation is that the activation of YAP/TAZ alleviates the dependency of cell proliferation on oncogenic KRAS signaling, suggesting that YAP/TAZ could be a downstream mediator of KRAS function in some contexts [34]. In this regard, KRAS and its associated pathways have been reported to regulate YAP/TAZ stability and activity via both Hippo-dependent and Hippo-independent pathways [36, 38–40].

Isoprenylcysteine carboxylmethyltransferase (ICMT) is the last of the enzymes that catalysis the three step prenylation processing that posttranslationally modifies substrate proteins including RAS isoforms [41–44]. Numerous studies have shown the potential of targeting ICMT and its substrates in cancers [43, 45–51]. However, little is known about whether ICMT function sustains cancer cell stemness properties in KRAS-driven cancers. In this study, we provide compelling evidence that supports a previously unidentified role of ICMT in the regulation of TAZ degradation via modulating the function of mutant KRAS and its downstream RAF–MEK signaling to support cancer cell self-renewal.

## Results

### ICMT suppression reduces the self-renewal ability of MiaPaCa2 pancreatic and MDA-MB231 breast cancer cells

**Fig. 1:**
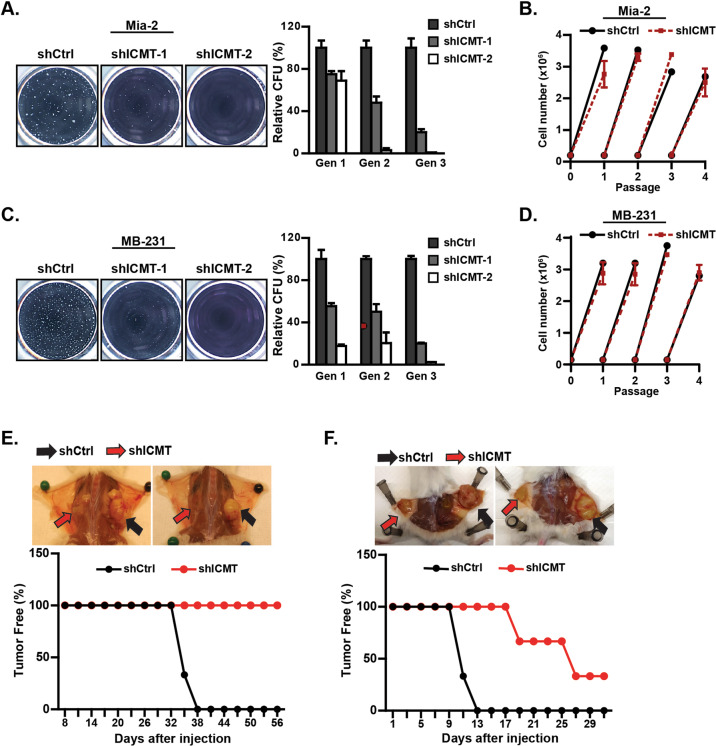
Suppression of ICMT abolishes the self-renewal ability of mutant KRAS-driven pancreatic and breast cancer cells. **a**, **c** Sphere formation study. MiaPaCa2 pancreatic (**a**) and MDA-MB231 breast (**c**) cancer cells were infected with lentivirus expressing either control shRNA or two ICMT-targeting shRNAs, followed by serial replating sphere formation studies. The images of the spheres were obtained after the third plating growth (3rd generation spheres, left panel). The sphere counts from three technical repeats for each replating (generation 1, 2, and 3) were analyzed by OpenCFU and Prism5, and presented as bar graphs (right panels). Each sphere formation assay was performed with three technical repeats; and the experiments were repeated in three biological repeats with similar results. ICMT expression levels, assessed by RT-qPCR in the two cell lines with and without ICMT knockdown, are shown in Supplementary Fig. S1A. Proliferation rates under normal adherent growth conditions of MiaPaca2 (**b**) and MDAMB-231 (**d**) cells expressing either control or ICMT shRNA. Shown are the changes in total cell numbers during 3 days of culturing for each passage vs. the passage number, with 3-day intervals between each passage. **e**, **f** In vivo tumor formation study. MiaPaca2 (**e**) and MDA-MB231 (**f**) cells (80,000 each) expressing either control or ICMT-targeting shRNA were injected subcutaneously into NOD-SCID mice (*n* = 10 tumors for each group). The mice were observed every 2 days until the largest tumor reach the volume limit set by the IACUC protocol, at which time all the mice were euthanized. The percentages of tumor-free mice were plotted (bottom panels) up to 56 days and 30 days for the tumors derived from MiaPaCa2 (**e**) and MDA-MB231 (**f**) cells, respectively.

To assess the role of ICMT in cancer cell self-renewal, we introduced into MiaPaCa2 and MDA-MB231 cells either control shRNA, or two independent shRNAs targeting ICMT that effectively reduced the ICMT expression (Supplementary Fig. S1A). These cells were assayed for sphere formation in the serial replating assay. At the end of each round of culturing, the spheres were photographed and then collected, cells separated, and reseeded for the subsequent round. The sphere numbers, particularly after the third seeding, represent self-renewal ability or stemness of the cancer cells. We observed a dramatic inhibition of sphere formation in MiaPaCa2 (Fig. 1a) and MDA-MB231 (Fig. 1c) cells expressing ICMT shRNA in comparison with the control group, which was especially evident in the third-generation culture. In addition to these two cell lines, similar reduction of sphere formation upon ICMT knockdown was observed in other KRAS mutant pancreatic cancer cell lines, including AsPC1 and PANC1 (Supplementary Fig. S1B and S1C).

The multi-passage sphere culture is well established as an assay for continuous self-renewal, i.e., stemness, of cancer cells [52–55]. However, we recognize that it is also useful to make the distinction between an effect on proliferation and the effect on stem cell renewal. To this end, we performed proliferation/viability assays on 2D adherent culture over many passages, alongside the three generation replating sphere formation assay for the whole duration. The cells for the proliferation assay were seeded from the same pool as those for sphere culture, grown to confluency, counted and reseeded, thus repeating the cycle. We have found that the proliferation rates under normal adherent growth condition are not affected by ICMT knockdown for either MiaPaCa2 (Fig. 1b) or MDA-MB231 (Fig. 1d) cells, in stark contrast with the 3rd generation sphere formation that was completely abolished by ICMT knockdown (Fig. 1a, c). These data enforce the conclusion that ICMT regulates stemness or self/renewal ability, but not proliferation in general.

A commonly used and reliable method to quantify the tumor inhibiting/stem cells and assess the self-renewal ability of cancer cells is in vivo tumor formation using a low number of cancer cells. Hence, instead of implanting 5 million cells for tumor formation as in previous studies [56–58], we injected 80,000 MiaPaCa2 (Fig. 1e) or MDA-MB231 (Fig. 1f) cells, expressing either control or ICMT-targeting shRNA, subcutaneously into the contralateral sides of groups of mice. Cells expressing ICMT-targeting shRNA showed significant delay or prolonged latency in tumor formation, which is presented as the fraction of mice that remains tumor free at a given time point (Fig. 1e, f). We also quantified tumor forming ability using the so-called limiting dilution assay, which involves reducing the number of cells for implantation to the point where no tumors form. By determining the tumor-initiating frequency following injection of 80,000, 20,000, and 5,000 control or ICMT knockdown cells into mice, we were able to approximate the tumor-initiation frequency of control cells or those with 85–90% ICMT knockdown in MiaPaCa2 at 1/33,000 vs. 1/130,000, and in MDA-MB231 at 1/3,000 vs. 1/42,000 cells (Supplementary Table 1). Together, the in vitro and in vivo observations strongly support an essential role of ICMT in maintaining cancer cell self-renewal ability/stemness properties.

### ICMT suppression enhances the efficacy of chemotherapeutic agents in inhibiting the self-renewal ability of MiaPaCa2 pancreatic and MDA-MB231 breast cancer cells

**Fig. 2:**
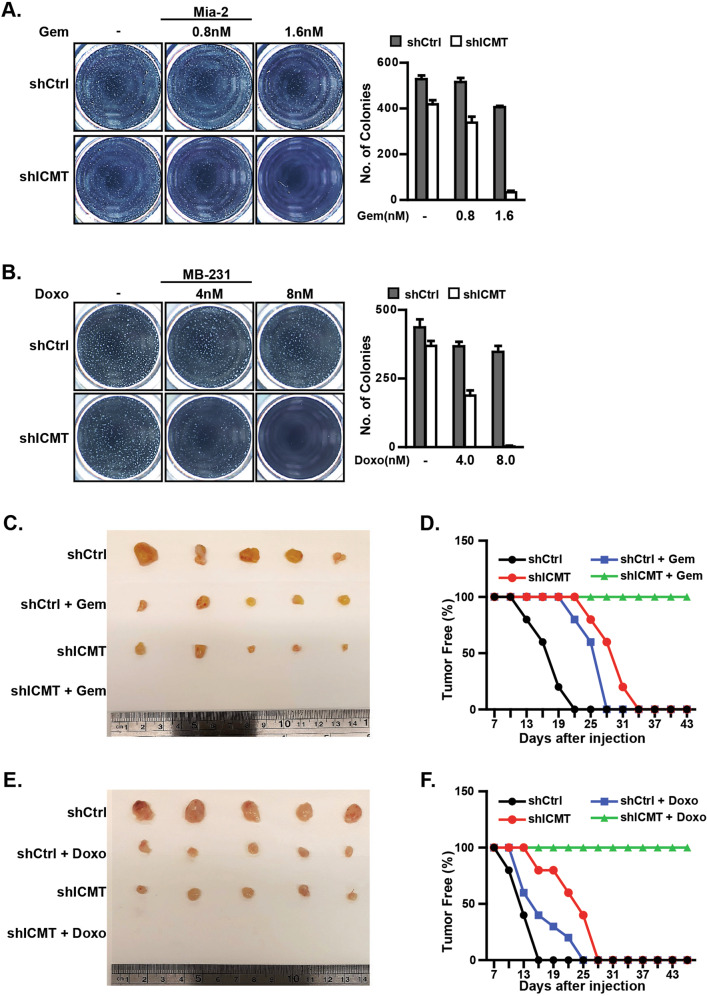
Moderate ICMT knockdown enhances the effect of gemcitabine and doxorubicin in inhibiting the self-renewal and tumorigenic capacities of MiaPaCa2 and MDA-MB231 cells, respectively. **a** MiaPaCa2 cells expressing either control or ICMT-targeting shRNA were cultured under the sphere forming conditions and treated with 0, 0.8, or 1.6 nM of gemcitabine as indicated. The sphere forming ability was assessed in three consecutive platings. The sphere images (left) were taken at the end of the third plating (3rd generation). The bar graph (right) presents the quantitation of sphere numbers from three technical repeats at the end of the 3rd plating; gray bar: control shRNA, white bar: ICMT-targeting shRNA. **b** Studies were performed as in **a** but with MDA-MB231 cells and that the chemotherapeutic agent used was doxorubicin at 0, 4, and 8 nM in combination with either control shRNA or that targeting ICMT. Data are presented as in **a**. For both panels, each sphere formation assay was performed with three technical repeats, and the experiments were repeated in three biological repeats with similar results. **c**, **d** MiaPaCa2 cells (80,000) expressing either control or ICMT-targeting shRNA were injected subcutaneously into contralateral flanks of NOD-SCID mice. Seven days after tumor implantation, the mice were divided into treatment and vehicle groups (*n* = 10 tumors each group); the treatment group received 150 mg/kg of gemcitabine twice a week. Tumor formation was monitored over the course of the study. At the end of the study, the mice were euthanized and the tumor excised and imaged (**c**). The percentage of tumor-free mice was plotted vs. treatment duration (**d**). **e**, **f** The same tumor formation study as shown in **c** and **d** was performed, except with MDA-MB231 cells and that the treatment group received doxorubicin at 1.5 mg/kg three times a week.

It has been widely recognized that the stem cell-like populations in cancer are highly resistant to chemotherapeutic agents [59]. This has been consistently shown for breast and pancreatic cancers, in which treatment failures are often attributed to the stem cell populations [1, 5, 60]. Hence, we assessed whether moderate ICMT inhibition would enhance the ability of gemcitabine and doxorubicin, frontline chemotherapies for pancreatic and breast cancer treatment, respectively, to eliminate CSCs. For this assessment, we lowered the titer of shRNA-carrying lentivirus to reduce the level of ICMT knockdown so that we could observe a combination effect. Prior to the combination study, we evaluated dose responses of the impact of gemcitabine and doxorubicin on MiaPaCa2 and MDA-MB231, respectively, as single agent in sphere formation cultures. Using the doses of the gemcitabine that have minimal impact in combination with moderate ICMT knockdown (<80% by qPCR), we observed that, while each alone only moderately reduced the number of spheres of MiaPaCa2 cells, the combination of the two abolished sphere formation (Fig. 2a). In MDA-MB231 breast cancer cells, similar impact was observed with the combination of doxorubicin and ICMT knockdown (Fig. 2b). It is important to note that the concentrations of gemcitabine and doxorubicin used in the current study are significantly lower than those reported in other in vitro cell studies [61, 62], suggesting that ICMT inhibition holds high promise in enhancing the efficacy of these chemotherapeutic agents without eliciting significant toxicity.

We next evaluated the combination effect of ICMT knockdown and chemotherapy in vivo in the mouse model. For these studies, NOD-SCID mice were grouped into the treatment and vehicle control groups; each mouse in either group was subcutaneously implanted with cancer cells expressing control shRNA on one side, and the cells expressing ICMT-targeting shRNA on the other side of the flank. Seven days after the implantation, the mice were dosed with either vehicle or the drug. The dosing regimens were 150 mg/kg, twice a week by IP for gemcitabine and 1.5 mg/kg, three times a week IP for doxorubicin, respectively [1, 5]. For MiaPaCa2 tumors, we observed some reduction in tumor size and delay in tumor formation in either the ICMT knockdown or gemcitabine treatment alone groups. Remarkably, the combination completely abolished tumor formation (Fig. 2c, d). Similar results were obtained with MDAMB-231 tumors; while either doxorubicin or moderate ICMT knockdown alone delayed tumor initiation and reduced tumor size, the combination completely inhibited tumor formation (Fig. 2e, f). Taken together, these results indicate that ICMT inhibition enables common chemotherapeutic drugs to eliminate cancer cell self-renewal, a much desired efficacy for these agents. This attribute of ICMT inhibition in targeting cancer stem cell population, either alone or in combination, can be potentially exploited for effective cancer therapy against aggressive and advanced tumors.

### Suppression of ICMT inhibits cancer stem cell self-renewal by enhancing TAZ, but not YAP, protein degradation

**Fig. 3:**
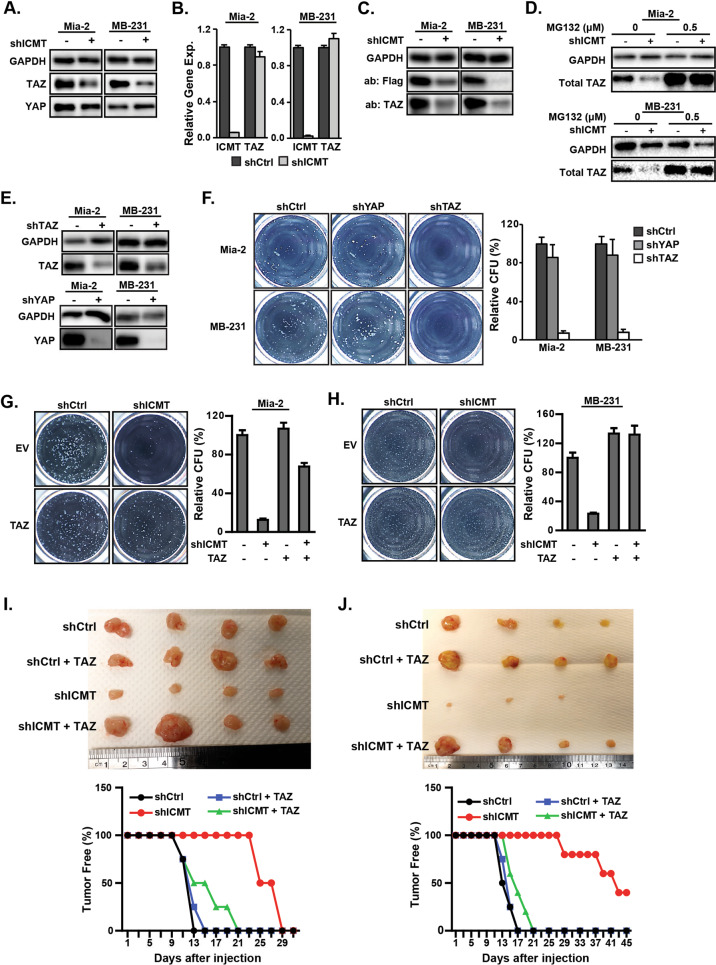
ICMT regulates cancer cell self-renewal/stemness via the control of TAZ protein level. Suppression of ICMT reduces the TAZ protein (**a**), but not transcript (**b**) levels in MiaPaCa2 and MDA-MB231 cells. **a** The levels of TAZ and YAP proteins were assessed by immunoblot analysis. **b** RT-qPCR analysis for the relative gene expression levels of TAZ and ICMT. **c** Immunoblot analysis for both endogenous TAZ and the introduced Flag-tagged TAZ protein levels in MiaPaCa2 and MDA-MB231 cells in the presence or absence of ICMT knockdown. **d** Immunoblot analysis of TAZ protein levels in MiaPaCa2 (top) and MDA-MB231 (bottom) cells, with or without ICMT knockdown, following 24 h of treatment with the indicated concentrations of MG132, a proteasome inhibitor, or vehicle control. **e** Immunoblot analysis showing shRNA knockdown of TAZ (top) and YAP (bottom) in MiaPaCa2 and MDA-MB231 cells. **f** Impact of TAZ and YAP knockdown on sphere forming ability. Left: images from the third replating of cells expressing control or TAZ-targeting shRNA; right: quantification of the sphere numbers using OpenCFU and Prism. The data presented are from the analysis of three technical repeats of the same study, which was repeated three times with similar findings. **g**, **h** Ectopic expression of TAZ rescues the sphere formation ability of MiaPaCa2 and MDA-MB231 cells. **g** MiaPaCa2 cells expressing TAZ or control vector were infected with lentivirus coding either control shRNA or ICMT shRNA; the cells were then seeded for the sphere formation assay. Left: images of spheres formed after growth of the 3rd plating; right: bar graph presenting the quantification of sphere numbers from three technical repeats of the study. **h** The TAZ rescue studies as in **g** were performed on MDA-MB231 cells. All in vitro studies in **a**–**g** were repeated three times with similar results. Tumor formation abilities were studied on the same MiaPaCa2 (**i**) and MDA-MB231 (**j**) cells similarly prepared as in **g** and **h**, respectively. For each study, 80,000 cells were injected subcutaneously into NOD-SCID mice; *n* = 10 tumors for each condition. Tumor formation was monitored through the course of the study until the control tumors reached the size limit set by IACUC protocol, whereupon the mice were euthanized and tumors excised. The top of both panels show the images of the excised tumors from the respective groups; in the bottom panels the percentage of tumor-free mice through the course of the study are plotted.

The essential role of ICMT in the self-renewal of cancer cells has not been previously reported. Hence, the molecular events underlying this ICMT function need to be defined. A number of pathways, including the HIPPO-YAP/TAZ signaling, have been linked to the stem cell-related characteristics [12, 19]. While examining the molecular changes associated with stemness, we have observed that the level of TAZ protein, but not its well-known paralog YAP, was robustly and consistently downregulated upon ICMT knockdown in both MiaPaCa2 and MDA-MB231 cells (Fig. 3a). RT-PCR analysis, however, showed no reduction of TAZ transcript level associated with ICMT knockdown (Fig. 3b), which suggests that the impact of ICMT silencing on TAZ protein level is posttranscriptional. To further the evaluation, we expressed Flag-TAZ fusion protein from a retroviral vector in both control and ICMT knockdown cells to observe whether the fusion TAZ protein produced from different promoter is also subjected to ICMT regulation. Indeed, we observed that both the endogenous TAZ and the Flag-TAZ levels were similarly reduced by ICMT knockdown, supporting the posttranscriptional regulation model (Fig. 3c). To evaluate whether proteasome degradation plays a role in ICMT regulation of TAZ protein level, we assessed the impact of treatment by MG132, a general proteasome inhibitor, on TAZ levels in control and in ICMT knockdown cells. As expected, MG132 treatment elevated the basal level of TAZ in both cell lines, consistent with the notion that proteasome degradation is an important factor in TAZ protein metabolism (Fig. 3d). More interestingly, we observed that MG132 equalized the TAZ levels between the control and ICMT knockdown cells, supporting the notion that ICMT regulates the proteasome degradation of TAZ (Fig. 3d).

Despite the extensive evidence in support of the roles of TAZ/YAP in cancer cell stemness, it is essential to assess the relevance of TAZ protein in maintaining stemness in specific cells of interest, which are MiaPaCa2 and MDA-MB231 in this case. It is particularly interesting to study whether TAZ and YAP function differently in this account, given the finding that ICMT preferentially regulates TAZ stability. To this end, we knocked down either TAZ or YAP in the two cancer cell lines by shRNAs (Fig. 3e). Moderate reduction of TAZ abolished tumor sphere formation, confirming the indispensable role of TAZ in supporting self-renewal in both cell lines; in contrast, YAP knockdown, failed to significantly reduce sphere formation (Fig. 3f). The comparative knockdown study suggests that MiaPaCa2 and MDA-MB231 cancer cells are less dependent on YAP for self-renewal/stemness.

Having obtained evidence on the importance of TAZ in these cancer cell lines, we further evaluated the role of TAZ acting downstream of ICMT in supporting cancer stemness. To this end, we performed rescue experiment in ICMT knockdown cells by ectopic expression of TAZ. Enforced expression of TAZ in both MiaPaCa2 and MDA-MB231 cells restored the sphere formation ability that was reduced by ICMT knockdown (Fig. 3g, h; see also Supplementary Fig. S2 for expression data). Consistent with the sphere formation result, enforced expression of TAZ also rescued xenograft tumor formation ability of ICMT knockdown cells (Fig. 3i, j). As observed in the earlier experiments (see Figs. 1 and 2), ICMT suppression resulted in longer latency for tumor formation, which was nearly completely reversed by the expression of TAZ. Worth noting, simply restoring TAZ to endogenous levels was sufficient to restore sphere and tumor formation to that of control cells, suggesting a physiologically relevant rescue. Taken together, these findings support a role for ICMT in regulating TAZ protein levels, and that this property is important in the self-renewal ability of cancer cells. It is important to note again that, in the same cancer cells, suppression of ICMT has little effect on YAP protein. These findings not only suggest that TAZ and YAP are subject to different regulatory mechanisms, but also that TAZ may have a more prominent role in the self-renewal of KRAS-driven cells.

### Mutant KRAS, a substrate of ICMT, is the major mediator for ICMT regulation of TAZ and self-renewal

As a critical enzyme in post-translation modification, ICMT has been found to be important for the functions of many of its substrate proteins [63–65]. To investigate the mechanism of ICMT regulation of TAZ and cancer cell self-renewal, we focused on ICMT substrates that have known roles in tumorigenesis. Since the cell lines used in the studies, MiaPaCa2, MDA-MB231, AsPC1, and PANC1, all harbor activating KRAS mutations, we evaluated whether mutant KRAS was involved in the regulation of TAZ protein levels by ICMT. It is worthwhile to point out that, despite the recognition of the importance of mutant RAS in tumorigenesis, the manner of RAS regulation of stemness is in need of better understanding; this is highlighted by our specific finding that RAS differentially regulates TAZ, but not YAP, to support cancer cell self-renewal.

**Fig. 4:**
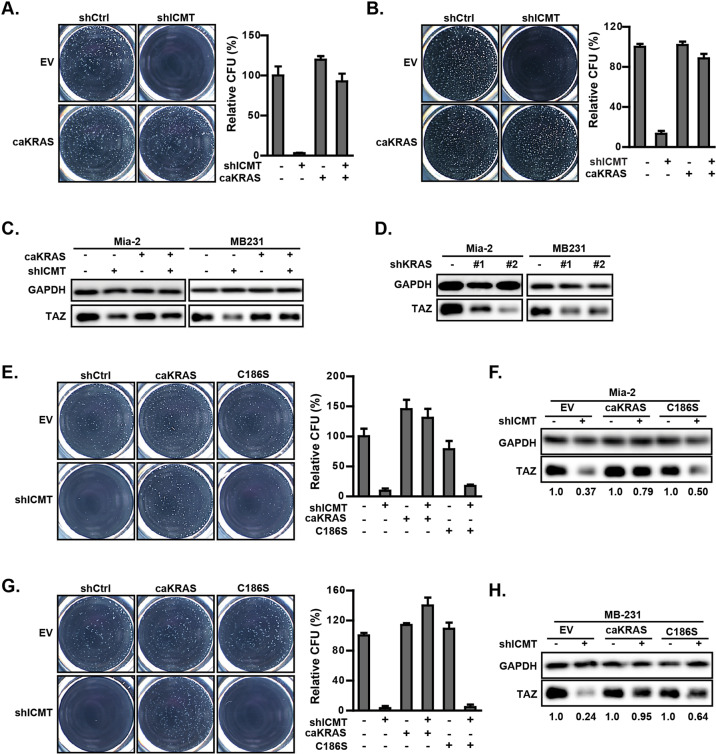
Expression of constitutively active KRAS rescues TAZ protein level and the self-renewal ability of MiaPaCa2 and MDA-MB231 cells expressing ICMT shRNA. Overexpression of constitutively active mutant KRAS (CA-KRAS) rescues the sphere formation ability lost upon ICMT silencing in MiaPaCa2 (**a**) and MBA-MB231 (**b**) cells. Cells were subject to the manipulations as indicated by expressing either control or ICMT-targeting shRNA, with or without concurrent expression of CA-KRAS. Left: images of spheres formed after the third plating; right: quantification of sphere numbers from three technical repeats of the third plating. **c** Immunoblot analysis of TAZ protein levels in the cells used for **a** and **b**. **d** Immunoblot analysis of TAZ protein levels in MiaPaCa2 and MDA-MB231 cells expressing either control shRNA or that targeting KRAS as indicated. **e** Sphere formation study on MiaPaCa2 cells, either in the presence or absence of ICMT knockdown, which concurrently express CA-KRAS, CA-KRAS(C186S), or vector control, respectively as indicated. Left: images of the third-generation spheres; right: quantitation of sphere numbers using OpenCFU and Prism5 software. **f** Immunoblot analysis of TAZ protein in the MiaPaCa2 cells used for the study in **e**. The relative quantities of TAZ protein between control shRNA and ICMT shRNA expressing cells are analyzed and presented below the blot. **g**, **h** Similar studies as in **e** and **f** were performed using MDA-MB231 cells. All studies have been repeated three times with similar results.

KRAS knockdown by two different targeting shRNAs abolished tumor sphere formation in MiaPaCa2 and MDA-MB231 cells, confirming the importance of KRAS in the self-renewal of these cells (Supplementary Fig. S3A and S3B). In contrast, knockdown of another ICMT substrate RHOA, which is also often implicated in tumorigenesis, failed to significantly impact the sphere formation of the two cell lines (Supplementary Fig. S3C and S3D). We then investigated whether overexpressing KRAS-G12V, hereafter referred to as CA-KRAS (constitutively active KRAS), could increase the TAZ protein level and rescue the sphere forming ability in the ICMT knockdown cells. Indeed, overexpression of CA-KRAS negated the effect of ICMT knockdown and restored the sphere formation in both cell lines (Fig. 4a, b). More relevant to the current study, CA-KRAS expression increased TAZ protein levels in ICMT knockdown cells while exerting little effect on the baseline level of TAZ in control cells (Fig. 4c). Careful study of TAZ levels in these experiments revealed that it is tightly regulated; the overexpression of either TAZ itself (Supplementary Fig. S2) or KRAS (Fig. 4c) never led to the over-baseline TAZ level, providing confidence for the physiological relevance for the rescue studies and accentuating the importance of ICMT impact on TAZ. Further evidence supporting the role of mutant KRAS in the control of TAZ levels was obtained from shRNA knockdown of KRAS, which demonstrated a significant reduction of TAZ protein in both cell lines (Fig. 4d).

The evidence detailed above suggests that ICMT regulates TAZ levels via its functional carboxylmethylation of KRAS. To seek further evidence to support the importance of the C-terminal posttranslational modification in the function of KRAS, we compared the rescuing ability of CA-KRAS with that of its C-terminal mutant—CA-KRAS(C186S)—that lacks the prenylation attachment site cysteine. Aa assessed by sphere formation, CA-KRAS(C186S), had dramatically reduced ability compared with CA-KRAS to rescue the sphere formation ability of ICMT knockdown cells (Fig. 4e, g). Consistently, CA-KRAS(C186S) also failed to effectively rescue the TAZ protein level, in contrast to CA-KRAS (Fig. 4f, h). The comparison between CA-KRAS(C186S) and CA-KRAS supports the notion that the C-terminal modification is important for the function of KRAS in the regulation of TAZ and cancer cell stemness.

**Fig. 5:**
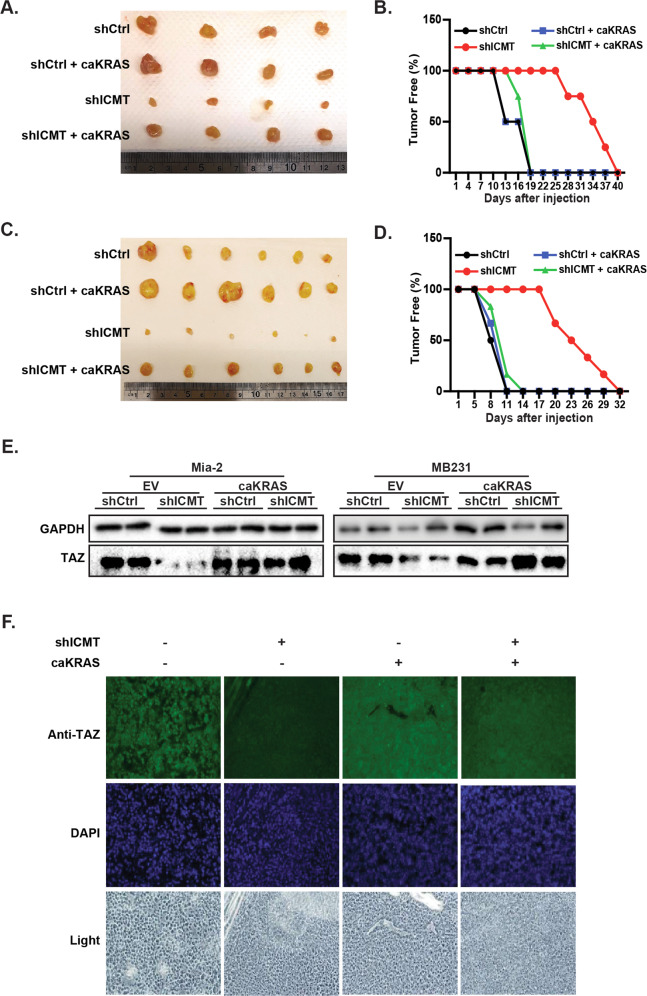
Expression of constitutively active KRAS restores both TAZ protein levels and the abilities to form xenograft tumors of MiaPaCa2 and MDA-MB231 cells with silenced ICMT. Tumor formation study using MiaPaCa2 (**a**, **b**) and MDA-MB231 (**c**, **d**) cells. For this study, 80,000 cancer cells either expressing CA-KRAS or control vector, in the presence or absence of ICMT knockdown, were injected subcutaneously into NOD-SCID mice; *n* = 10 tumors were implanted for each cell group. The mice were sacrificed and tumors excised when any tumor reached size limit set by the IACUC protocol. The images of the tumors derived from MiaPaCa2 and MDA-MB231 cell are shown in **a** and **c**, respectively. The percentages of tumor-free mice over the course of the experiment are plotted in **b** and **d** for the study groups of **a** and **c**, respectively. **e** Immunoblot analysis of TAZ protein levels for the tumor samples in **a** and **c**, respectively. **f** Immunofluorescent analysis of TAZ protein using the tumor samples from **a**.

We also evaluated whether enforced expression of CA-KRAS could rescue the in vivo tumor forming ability that is lost upon ICMT knockdown. MiaPaCa2 (Fig. 5a, b) and MDA-MB231 (Fig. 5c, d) cells, with and without CA-KRAS expression and in the presence or absence of ICMT-targeting shRNA, were injected subcutaneously into SCID mice. Consistent with the in vitro data, cells expressing CA-KRAS were able to overcome the effect of Icmt shRNA and regained the tumor forming ability to the level of control cells. Similar to that observed in the in vitro study, expression of CA-KRAS reversed the reduction in TAZ protein levels resulting from ICMT knockdown, as assessed by tumor sample immunoblot (Fig. 5e) and immunohistochemistry analysis of the tumor tissue (Fig. 5f).

### RAF–MEK signaling is the major downstream pathway for KRAS regulation of TAZ and cancer cell self-renewal

**Fig. 6:**
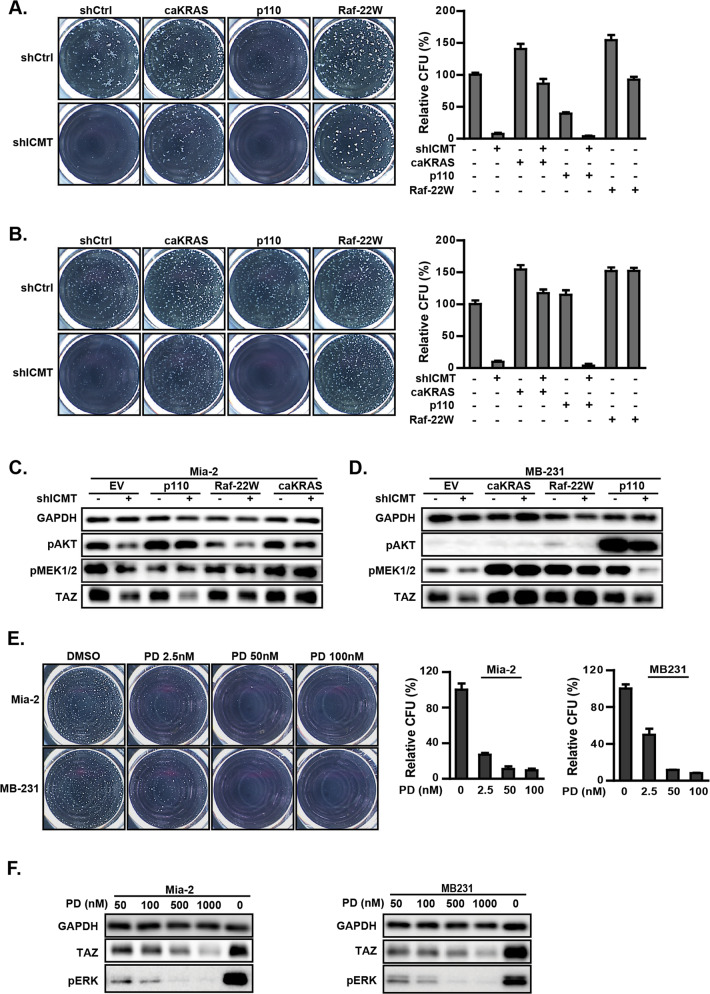
ICMT regulates cancer cell self-renewal/stemness via KRAS and its downstream effectors RAF and MEK. **a** Sphere formation assay on MiaPaCa2 cells expressing either control shRNA or ICMT-targeting shRNA, with concurrent expression of CA-KRAS, the PI3K catalytic subunit p110, the kinase active RAF-22W, or the empty vector control as indicated. The images of the spheres formed are shown on the left side; the quantifications of the spheres, using OpenCFU and Prism5 software on three technical repeats, are plotted on the right side. **b** The same experiment was performed as shown in **a** but with MDA-MB231 cells. **c**, **d** Immunoblot analysis of the same cells used for **a** and **b** to show the TAZ levels and the engagement of pMEK downstream of RAF and pAKT downstream of p110, as the result of the expression of CA-KRAS, RAF-22W, or p110. **e** Sphere formation assays on MiaPaCa2 and MDA-MB231 cells treated with increasing concentrations of MEK inhibitor PD184352. The images of the spheres formed are shown on the left side; the quantification to calculate the means and standard deviations, using OpenCFU and Prism5 software, on three technical repeats, are plotted on the right side. **f** Immunoblot analysis for TAZ and pERK levels in MiaPaCa2 and MDA-MB231 cells treated with the indicated concentrations of PD184352. All studies have been repeated three times with similar results.

Mutant RAS is involved in many cancer-related processes. As such, RAS has been found to engage multiple downstream signaling pathways, among which the RAF–MEK and PI3K–AKT axes are the most studied [66, 67]. To assess signaling effectors downstream of KRAS in the regulation of self-renewal and TAZ levels in MiaPaCa2 and MDA-MB231 cancer cells, a constitutively active RAF mutant, RAF-22W [68], and membrane localizing PI3K catalytic subunit (p110α-CAAX) [69] were introduced to activate the RAF–MEK and PI3K/AKT pathway, respectively. The effect of these pathway-specific activators on rescuing the self-renewal ability of the cells expressing ICMT-targeting shRNA was assessed by sphere formation assays; cells expressing CA-KRAS were used as positive controls. Consistent with the results noted above, CA-KRAS expression restored the sphere formation ability lost upon ICMT knockdown in both cell lines (Fig. 6a, b). Interestingly, we found that, only RAF-22W, but not p110, rescued the sphere formation (Fig. 6a, b), which suggests that, in these two aggressive human cancer cell lines, MiaPaCa2 and MDA-MB231, ICMT regulates mutant KRAS function to maintain TAZ level and cancer stemness/self-renewal mainly through the RAS–RAF signaling pathway. As expected, introduction of RAF-22W and p110α resulted in the elevation of phosphorylated MEK and AKT, respectively, demonstrating the anticipated engagement of RAF–MEK and PI3K signaling (Fig. 6c, d). RAF-22W did not rescue the pAKT level and, vice versa, p110α did not increase pMEK levels in ICMT knockdown cells (Fig. 6c, d), which demonstrates well-separated signaling mechanisms allowing assessment of their respective functional impact. Consistent with previous studies, CA-KRAS expression leads to significant activation of both pRAF–pMEK and pAKT signatures (Fig. 6c, d).

Given the importance of RAS–RAF–MEK and RAS–PI3K–AKT signaling in cancer, we sought to confirm their differential regulation of TAZ by pharmacological inhibition with target-specific inhibitors—PD184352 (a MEK inhibitor), triciribine (an AKT inhibitor), and rapamycin (a mTOR inhibitor). For each of these inhibitors, we first identified a range of drug concentrations that only inhibited the intended target but not the other pathways. We then studied the impact of each inhibitor on TAZ level and sphere formation. Consistent with the RAF-22W rescue results, PD184352 inhibited sphere formation of both cell lines in a dose-dependent fashion (Fig. 6e). Further, the level of TAZ reduction correlated with that of pERK in a PD184352 concentration-dependent manner (Fig. 6f). Consistent with the notion that TAZ, but not YAP, plays important roles in regulating self-renewal in these cell lines (Fig. 3f), PD184352 treatment, even at high concentrations, had minimal effect on YAP levels in contrast to its effect on TAZ protein (Supplementary Fig. S4). Consistent with the p110 overexpression results, we found that, at concentrations that sufficiently inhibited respective signaling, neither sphere forming ability nor TAZ levels were significantly affected by the AKT inhibitor triciribine or the mTOR inhibitor rapamycin (Supplementary Fig. S5).

In conclusion, the expression of activated RAS effectors and the inhibitor studies support the notion that RAS–RAF–MEK signaling positively regulates TAZ protein levels in support of cancer stem cell self-renewal, and that ICMT function is essential for KRAS control of RAF–MEK signaling activation, TAZ protein level and cancer stemness. It is interesting that the other most-studied RAS downstream effector, PI3K, is not involved in the positive regulation of TAZ, in contrast to some earlier observations [70]. Remarkably, while significant inhibition of RAS–RAF signaling reduced TAZ levels and sphere formation, it does little to YAP, providing additional evidence that there are distinct differences in YAP/TAZ function and regulation, particularly by RAS–RAF signaling.

### ICMT inhibitors have similar effects as ICMT shRNA in reducing TAZ protein and abolishing tumor sphere formation

**Fig. 7:**
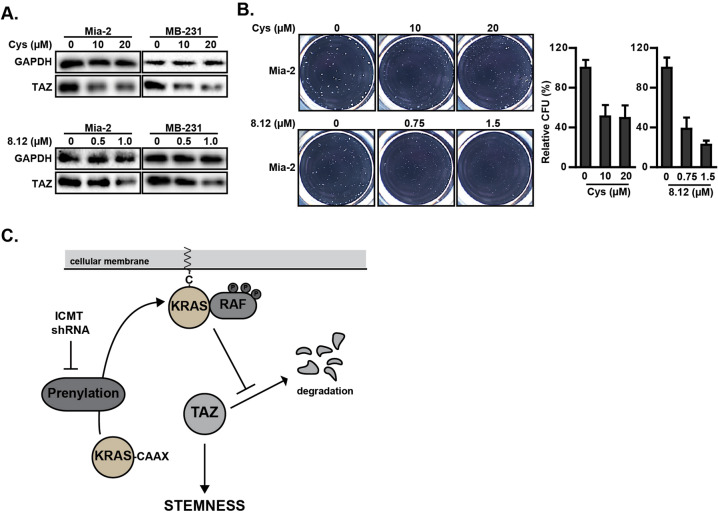
Inhibition of ICMT reduces TAZ protein level. **a**, **b** The impact of ICMT on TAZ protein and tumor sphere formation was evaluated using two different ICMT small molecule inhibitors—cysmethynil and cpd8-12. **a** Immunoblot analysis of TAZ protein levels in MiaPaCa2 and MDA-MB231 cells after the treatment by either control vehicle or the indicated concentrations of cysmethynil (top) or cpd8-12 (bottom). **b** MiaPaCa2 tumor spheres formed under the treatment of either vehicle or the indicated inhibitor. The images of the spheres are shown on the left side; the quantifications to calculate the means and standard deviations, using OpenCFU and Prism5 software, on three technical repeats are plotted on the right side of the panel. All experiments have been repeated three times with similar results. **c** Schematic model for the ICMT regulation of TAZ stability and cancer cell self-renewal via KRAS and MAPK signaling pathway.

So far, our data on the impact of ICMT suppression in downregulating TAZ protein levels in various cancer cell lines comes from genetic inhibition of ICMT. While these previously undescribed findings have potential therapeutic significance in identifying a path to suppress KRAS-driven cancer self-renewal, it would be helpful to determine whether pharmacological inhibition of ICMT can achieve the same effect. To this end, we made use of two small molecule inhibitors of ICMT, cysmethynil, and cpd8-12, that we have developed [51, 71]. Consistent with the shRNA results, we observed the dose-dependent reduction of TAZ protein in both cell lines treated with cysmethynil and cpd8-12 (Fig. 7a). Correspondingly, we also observed the dose-dependent reduction of tumor spheres by treatment with these ICMT inhibitors (Fig. 7b). These results simultaneously confirm the findings using genetic suppression of ICMT and also demonstrate the potentials of pharmacologically targeting ICMT in the treatment of KRAS-driven cancers via reduction of TAZ-dependent cancer stemness.

In summary, this study has uncovered a previously unrecognized role for ICMT in the regulation of TAZ levels mediated by the ICMT substrate KRAS and its downstream effector RAF. The impact on KRAS function through the inhibition of its modification by ICMT results in decreased RAF–MEK activation and subsequent decrease in TAZ protein, leading to the loss of self-renewal ability/stemness in KRAS-driven cancer cells (Fig. 7c). In a broad sense, this study underscores the importance of ICMT in the regulation of RAS–RAF signaling, which holds fundamental importance in cancer-specific functions and poses formidable therapeutic challenge.

## Discussion

CSCs play important roles in tumor recurrence and treatment failure. Although conventional chemotherapeutic agents are able to reduce the tumor bulk in short term, they are often less effective against the stem cell population, which is a major cause for relapse, metastasis, and mortality. Therefore, it is critical to find ways to eliminate cancer stem cell population, particularly in aggressive cancers such as pancreatic and triple-negative breast cancers. Noteworthy, mutant RAS-driven cancers are among the most difficult to manage and achieve long-term remission.

### Therapeutic implications

Mutant RAS proteins are major drivers for about one-third of human cancers; these RAS-driven cancers constitute the most fatal diseases due to the lack of effective targeted therapy. Despite recent development of inhibitors against KRAS(G12V) [72, 73], which accounts for a small fraction of RAS mutants, RAS remain extremely difficult to target. Abnormal activation of TAZ function leads to tumor formation and increased tumor stemness. As a transcription activator, TAZ is inherently challenging to target directly. Evidence from this study supports the critical role of ICMT in the negative regulation of both KRAS and TAZ function in supporting cancer cell self-renewal. By posttranslational carboxylmethylation, ICMT modulates KRAS function in engaging RAF–MEK signaling, which in turn affects TAZ stability and cancer stem cell self-renewal. The discovery of ICMT regulation of two fundamental regulators in cancer, RAS and TAZ, is exciting for the potential therapeutic utility of ICMT inhibition.

### PI3K signaling has little impact on TAZ stability in MiaPaCa2 and MDA-MB231 cancer cells

RAF–MEK and PI3K–AKT signaling are the two major KRAS downstream pathways. Previous studies suggest that PI3K, as well as RAF signaling, can positively regulate TAZ level and function [70, 74]. However, in contrast to these reports, our results, both from stimulatory studies of introducing active RAF and p110α and from pathway-specific inhibitor assessment, demonstrate that only RAF–MEK but not PI3K/AKT signaling regulates TAZ stability and self-renewal of MiaPaCa2 and MDA-MB231 cells. If anything, it appears that activation of PI3K pathway slightly suppressed the sphere formation in these cells. The discrepancy of the current finding and the prior reports underscores the complexity of TAZ regulation and the importance of thorough evaluation in specific cellular contexts.

### The difference between YAP and TAZ

In this study, TAZ was observed to be far more responsive to ICMT inhibition and KRAS–RAF signaling changes than its paralog YAP (Fig. 3a, b and Supplementary Fig. S5). We have also observed that YAP reduction has little effect, in contrast to that observed with TAZ reduction, on the self-renewal of these cancer cells (Supplementary Fig. S2C). Although often considered similarly regulated, YAP and TAZ proteins contain different structure elements that can be regulated differently. One of the most noticeable difference is that TAZ has C- and N-terminus degron phosphorylation sites, while YAP only has the C-terminal one that is subject to LATS-dependent phosphorylation [74, 75]. In our assessment, LATS activation status changes little upon ICMT knockdown and subsequent rescue by KRAS–RAF, suggesting a LATS-independent regulatory mechanism. Consistent with this notion, YAP is less responsive to both ICMT knockdown and MEK inhibition, pointing to a distinct regulatory mechanism for TAZ. Recent studies suggest that there are kinases other than LATS that are involved in phosphorylation of TAZ but not YAP [74]. While the identity and function of the kinase(s) need further evaluation, it is clear that TAZ and YAP levels and functions are subjected to both overlapping and differential regulations. The evidence of CA-KRAS and activated RAF rescuing the TAZ levels reduced by ICMT knockdown, and of the MEK inhibitor inducing robust TAZ, but not YAP, degradation lead us to speculate that whichever posttranslational modifications are involved in the differential regulation of TAZ, they are likely downstream effectors of RAF–MEK signaling.

## Materials and methods

### Cell culture, antibodies, and reagents

MiaPaCa2, MDA-MB231, Panc1, AsPC1, and HEK293T cell lines were obtained from American Type Culture Collection (ATCC) and mycoplasma-free. These cells were cultured in Dulbecco’s minimal essential medium (DMEM) from Nacalai (California, USA) supplemented with 10% v/v FBS and penicillin (100 U/mL)/streptomycin (100 μg/mL) from Hyclone (IL, USA). Antibodies for GAPDH (14C10, #2118), phospho-AKT Ser473 (#9271), phospho-ERK Thr202/Tyr204 (#9101), phospho-S6 Ser235/236 (#2211), YAP (#4912), phospho-YAP Ser127 (#4911), and phospho-LATS1 Ser909 (#9157) were from Cell Signaling Technology (MA, USA). Antibody for TAZ (#HPA007415) was from Sigma-Aldrich (MO, USA). Gemcitabine-HCl (#S1149) was obtained from Selleck Chemicals (TX, USA), while doxorubicin-HCl (#D-4000) from LC Laboratories (MA, USA). PD184352, Triciribine and Rapamycin were obtained from Sigma-Aldrich (MO, USA). The Cell viability was assayed by colorimetric based CellTiter 96^®^ AQueous One Solution Cell Proliferation kit (#G3581, Promega), per manufacture’s protocol.

### Transfection and generation of stable cell lines

Viruses used for shRNA and protein expression were produced in HEK293T cells [76]. Prior to infecting the cell line of interest, the virus-containing media from HEK293T cells was mixed with fresh 10% FBS DMEM at 50%v/v and with a final concentration of 10 μg/ml polybrene (#H9268, Sigma-Aldrich). The primers used for shRNA vector construction are listed in Supplementary Information.

### Quantitative real time PCR

RNA and cDNA were prepared using Tissue Total RNA Mini Kit (FATRK 001-2, Favorgen Biotech Corporation) and ReverTra Ace qPCR RT Master Mix (FSQ-201, Toyobo), respectively. Quantitative PCR was performed using Thunderbird SYBR qPCR Mix (Toyobo) using an Applied Biosystems 7900HT instrument. The primers used for PCR are listed in Supplementary Information.

### DNA constructs

For knockdown studies, we used the third-generation lentiviral shRNA expression system Lentilox 3.7 (pLL3.7). The design of shRNA primers and the cloning procedure can be found at: http://web.mit.edu/jacks-lab/protocols/pll37.htm. Briefly, the primers cover 19 base-pair of the target gene with the additional hairpin sequence of “ttcaagaga”. The target sequences for the genes of interest are listed in Supplementary Information. The primer pairs were first phosphorylated using T4 PNK (Thermo Scientific, USA), followed by annealing and cloning into pLL3.7. In the case of expression cloning, pBabe-Puro-myc-P110-CAAX [69] and pBabe-Puro-MEK1DD [77] were purchased from Addgene (MA, USA). pBabe-Puro-KRAS-G12V (CA-KRAS) was generated in the lab [45]. The coding sequence of TAZ (NM_015472) or Flag-TAZ was cloned in frame into XhoI/EcoRI sites of pMSCV-Blasticidin vector. pBabe-Puro-RAF1-22W was sub-cloned from pBabe-Neo-RAF1-22W plasmid (Addgene) [69].

### Tumor sphere formation assay

Cancer cells were seeded at 400 cells per well in low-adherent culture plates (0.32 cm^2^, Sigma) in 100 μl of 0.5%v/v methyl-cellulose (Sigma-Aldrich) in DMEM-F12 supplemented with B-27 and N-2 from Gibco (MD, USA). The tumor spheres were cultured until desired size before collected for imaging and/or replating [78, 79]. For subsequent replating, cells in the sphere were separated using StemPro^®^ Accutase^®^ Cell Dissociation Reagent (Gibco) and resuspended in sphere culturing medium as described above. OpenCFU software (Geissmann) was used to determine the sphere numbers from the microscopic images. For short-term tumor sphere culturing, cells were suspended at 100,000/ml in DMEM-F12 media supplemented with B-27 and N-2 and seeded in low-adherent six-well plates (Sigma-Aldrich), which were incubated for 3 days before being collected for molecular analysis.

### Xenograft mouse model

For tumor formation studies, 80,000 cells were harvested from adherent culture and suspended in DMEM containing 10% FBS and 20% matrigel (BD sciences). The cells were injected subcutaneously into the flanks of NOD-SCID-Gamma female mice that were 8–10 weeks old and weighed 18–20 g. The tumor cell implantation and drug treatment regimen are described in relevant figure legends. The tumor growth was monitored every 2 days. For each experimental group, at least ten tumors were included. Randomization and blinding are not required for these in vivo studies. The animals were handled in accordance with IACUC guidelines.

### Statistical analysis

All statistical analysis in this study was performed using GraphPad Prism software; data are presented as mean ± SD. To calculate the statistical significance, experimental groups were compared with the control group using Dunnet test one-way ANOVA to generate *P* values. Statistical significance was defined as *P* < 0.05.

## Supplementary information

Supplementary Information
