# Supplementary material for: Isoprenylcysteine carboxylmethyltransferase is required for the impact of mutant KRAS on TAZ protein level and cancer cell self-renewal
Source: Oncogene. 2020 Jun 19;39(31):5373–89. doi: 10.1038/s41388-020-1364-7 (PMC7391290; doi:10.1038/s41388-020-1364-7)
Supplement: Supplementary file 1 — Supplementary Information [file 41388_2020_1364_MOESM1_ESM.doc]

**Supplementary Information:**

The Supplementary Information contains two parts.

Part I contains Supplementary Figures and Table.

Part II contains tables listing the PCR primer and shRNA oligo sequences.

**I. Supplementary Figures and Table**

**Supplementary Fig. S1.** **(A)** ICMT expression level in cells expressing control or ICMT targeting shRNA; the cells are from the same study as in Fig 1A and 1C in the main figure. **(B, C)** ICMT knockdown leads to reduced sphere formation for AsPC-1 **(B)** and PANC-1 **(C)** pancreatic cancer cells. For both B and C, the left panels show the Icmt expression level in cells expressing control or ICMT-targeting shRNA; the middle panels show the images of sphere formation of the two conditions; the right panels show the quantification of three generations of sphere numbers.

**Supplementary Table 1.** Limiting dilution *in vivo* tumor formation efficiency study for MiaPaCa2 **(A)** and MDA-MB231 **(B)** cells. Control and ICMT-targeting shRNA were introduced into the parental cells for each of the cell lines. The indicated numbers of cells were subcutaneously implanted into contralateral flanks of the mouse. The mice were observed for tumor formation.


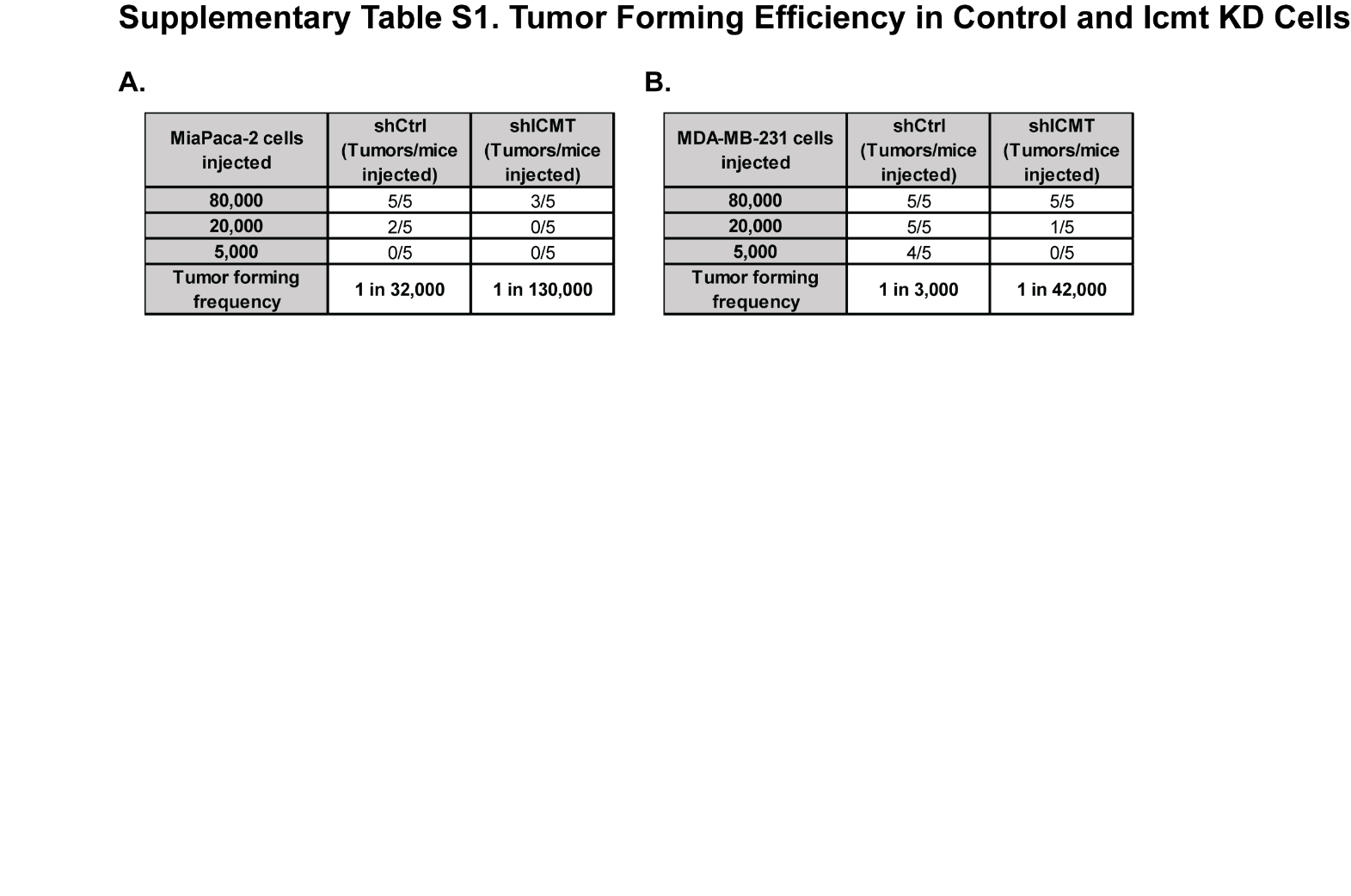


**Supplementary Fig. S2.** Ectopic expression of TAZ from introduced retroviral vectors replenish TAZ to baseline levels in ICMT knockdown MiaPaCa2 **(A)** and MDA-MB231 **(B)** cancer cells. Left panels in (A, B): RT-PCR analysis of ICMT and TAZ transcript levels; right panels in (A, B): immunoblot assessment of TAZ protein levels.


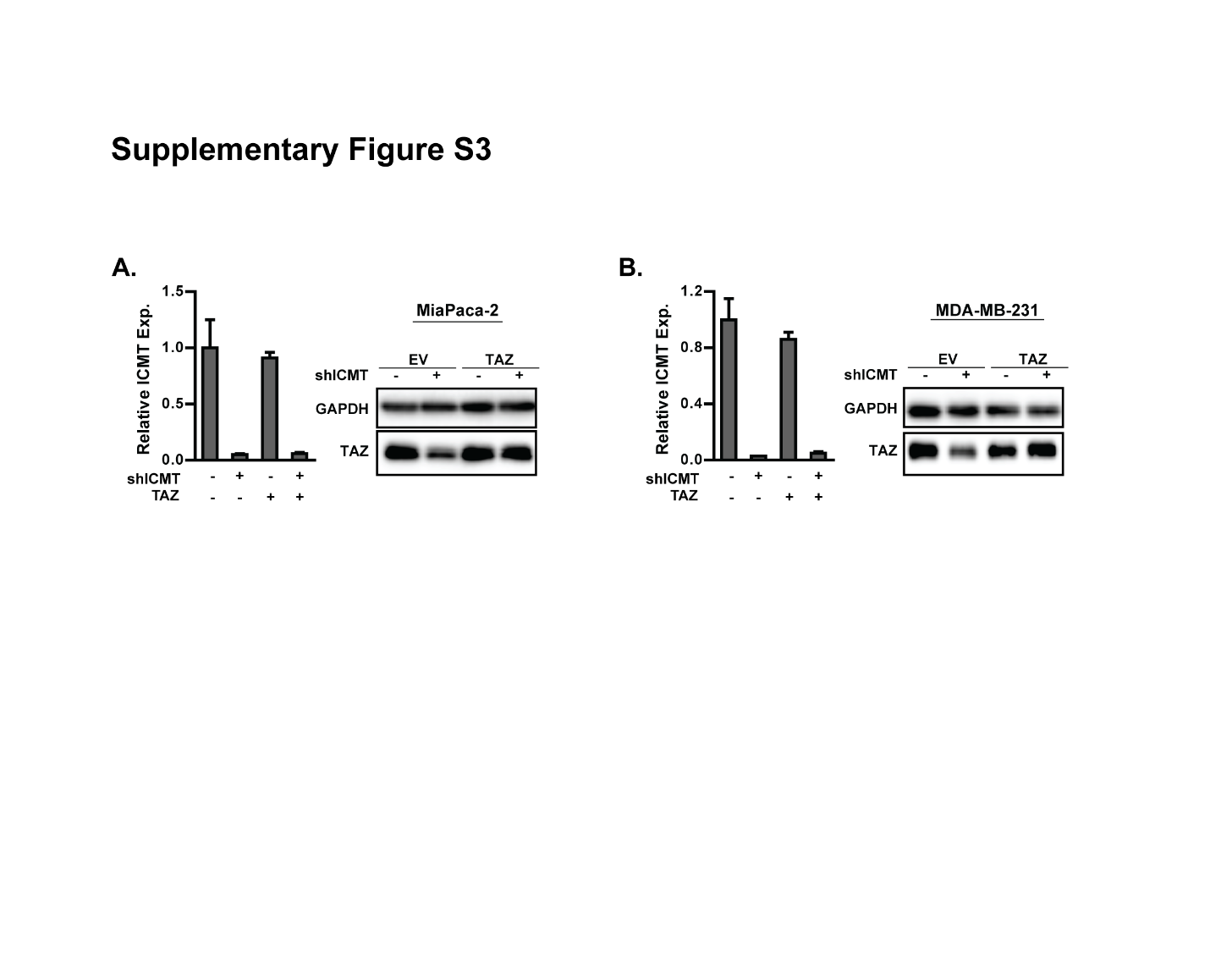


**Supplementary Fig. S3.** ShRNA knockdown of KRAS, but not RHOA, in MiaPaCa2 and MDA-MB231 cells abolishes tumor sphere formation. **(A, B)** Left panels: Images of tumor spheres from the third replating cultures of MiaPaCa2 (A) and MDA-MB231 (B) cells, with the expression of either control or KRAS-targeting shRNA. Right panels: The OpenCFU analysis of the sphere number quantification. **(C, D)** The same as in (A, B) except with RHOA-targeting shRNA. All data presented are from the three technical repeats of one experiment; all studies have been repeated three times with similar outcomes.


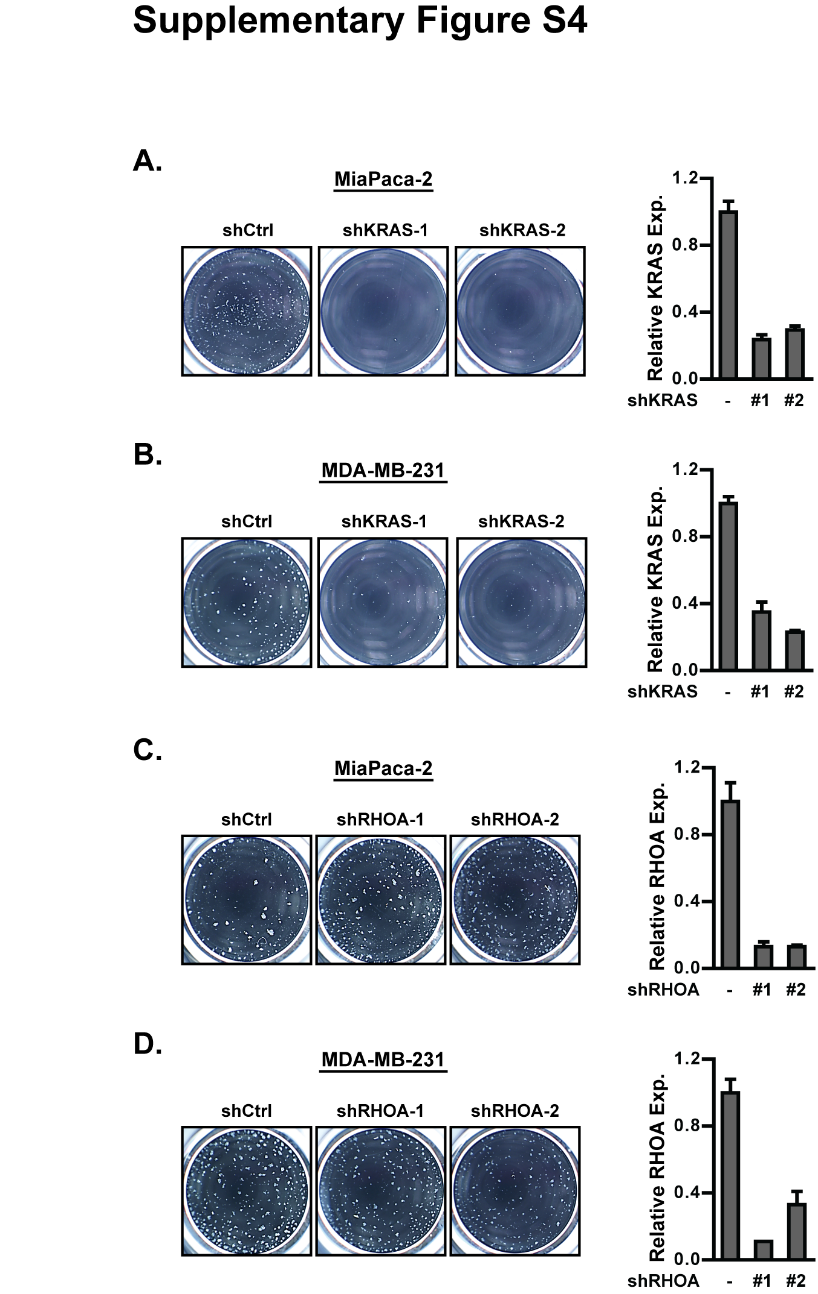


**Supplementary Fig. S4.** Immunoblot analysis of pERK, YAP and TAZ levels in MiaPaCa2 and MDA-MB231 cancer cells following treatment with MEK inhibitor PD184352.


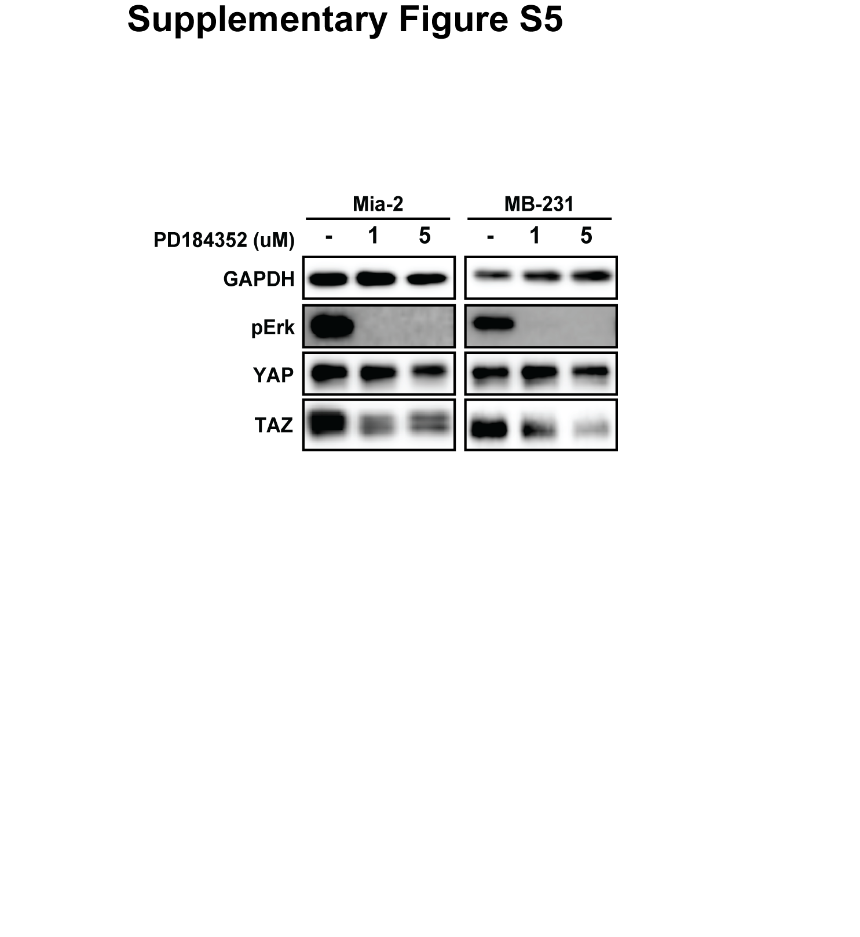


**Supplementary Fig. S5.** Treatment of MiaPaCa2 and MDA-MB231 cancer cells with AKT and mTOR inhibitors, triciribin and rapamycin, exerts no significant effects on either TAZ protein levels or sphere formation abilities. (A) Dose-response impact of mTOR inhibitor rapamycin on TAZ and its downstream effector pS6 levels. (B) Sphere formation efficiency of MiaPaCa2 and MDA-MB231 cells under rapamycin treatment. (C) Dose-response impact of AKT inhibitor triciribin on TAZ and its target pAKT levels. (D) Sphere formation efficiency of MiaPaCa2 and MDA-MB231 cells under triciribin treatment. The sphere counts for (B) and (D) are from three technical repeats analyzed by OpenCFU and Prism5, and presented as bar graphs.

**II. Primers and Oligo Sequences used in the paper**

**Table 1. Q-PCR primers used in the study.**

| **Gene** | **Primer sequence 5’🡪3’** |
| --- | --- |
| ICMT-F | GTTTCGGCATCCTTCTTACG |
| ICMT-R | CACTGTCAGGGCATAGCTGA |
| KRAS-F | GCAAGAGTGCCTTGACGATAC |
| KRAS-R | TCCAAGAGACAGGTTTCTCCA |
| KRAS_3’UTR-F | AGTGCCAGTCTTGGGCAAAA |
| KRAS_3’UTR-R | TGCATCAAGTCATGGGGCAT |
| TAZ-F | GGCTGGGAGATGACCTTCAC |
| TAZ-R | ATTCATCGCCTTCCTAGGGT |
| TAZ_3’UTR-F | TGACCTTACATTTCCTGGGC |
| TAZ_3’UTR-R | AGGCAATGATTAAACTGGCAAC |
| YAP-F | CCTTCTTCAAGCCGCCGGAG |
| YAP-R | CAGTGTCCCAGGAGAAACAGC |

**Table 2. shRNA oligo sequences.**

| **Gene** | **Target sequence 5’🡪3’** |
| --- | --- |
| shICMT-1 | GTCATTGTTCCACTATTCT |
| shICMT-2 | GGTTTCGGCATCCTTCTTA |
| shKRAS-1 | GTATCATTTGAGTGAATGT |
| shKRAS-2 | GAACCCTATCCAGTGGAAG |
| shTAZ-1 | GCCTCCATGACTCGTGCTC |
| shTAZ-2 | GGTACTTCCTCAATCACAT |
| shYAP-1 | GGCTGCCACCAAGCTAGAT |
| shYAP-2 | GGATGATGGATGCCATTCC |
